# Why Age and Aging Research Matters: A Chat With NIA Senior Leadership

**DOI:** 10.1093/geroni/igaa057.3010

**Published:** 2020-12-16

**Authors:** Marie Bernard

## Abstract

The National Institute on Aging (NIA) at the National Institutes of Health, Department of Health and Human Services, is the federally designated lead agency on aging research, and has supported significant research on aging as a life-long process. In the last five years, NIA experienced a tripling of its budget. Although much of this funding is targeted to Alzheimer’s disease (AD) and AD related dementias (ADRD) research, there was an increase in funds allocated to non-AD research in keeping with the overall growth of NIH. This symposium will provide a forum for exploration of the implications of the budget increases for the general research community. It will involve NIA’s senior staff discussing research priorities and programs supported by the Institute. A question-and-answer session will follow brief introductory remarks on current funding and future priorities and research directions of NIA.

